# Intranasal Administration of poly(I:C) and LPS in BALB/c Mice Induces Airway Hyperresponsiveness and Inflammation via Different Pathways

**DOI:** 10.1371/journal.pone.0032110

**Published:** 2012-02-15

**Authors:** Magnus Starkhammar, Susanna Kumlien Georén, Linda Swedin, Sven-Erik Dahlén, Mikael Adner, Lars Olaf Cardell

**Affiliations:** 1 Division of ENT Diseases, CLINTEC, Karolinska Institutet, Karolinska University Hospital, Stockholm, Sweden; 2 Unit for Experimental Asthma and Allergy Research, The National Institute of Environmental Medicine, Karolinska Institutet, Stockholm, Sweden; 3 The Centre for Allergy Reseach, Karolinska Institutet, Stockholm, Sweden; Massachusetts General Hospital and Harvard Medical School, United States of America

## Abstract

**Background:**

Bacterial and viral infections are known to promote airway hyperresponsiveness (AHR) in asthmatic patients. The mechanism behind this reaction is poorly understood, but pattern recognizing Toll-like receptors (TLRs) have recently been suggested to play a role.

**Materials and Methods:**

To explore the relation between infection-induced airway inflammation and the development of AHR, poly(I:C) activating TLR3 and LPS triggering TLR4, were chosen to represent viral and bacterial induced interactions, respectively. Female BALB/c or MyD88-deficient C57BL/6 mice were treated intranasally with either poly(I:C), LPS or PBS (vehicle for the control group), once a day, during 4 consecutive days.

**Results:**

When methacholine challenge was performed on day 5, BALB/c mice responded with an increase in airway resistance. The maximal resistance was higher in the poly(I:C) and LPS treated groups than among the controls, indicating development of AHR in response to repeated TLR activation. The proportion of lymphocytes in broncheoalveolar lavage fluid (BALF) increased after poly(I:C) treatment whereas LPS enhanced the amount of neutrophils. A similar cellular pattern was seen in lung tissue. Analysis of 21 inflammatory mediators in BALF revealed that the TLR response was receptor-specific. MyD88-deficient C57BL/6 mice responded to poly (I:C) with an influx of lymphocytes, whereas LPS caused no inflammation.

**Conclusion:**

*In vivo* activation of TLR3 and TLR4 in BALB/c mice both caused AHR in conjunction with a local inflammatory reaction. The AHR appeared to be identical regardless of which TLR that was activated, whereas the inflammation exhibited a receptor specific profile in terms of both recruited cells and inflammatory mediators. The inflammatory response caused by LPS appeared to be dependent on MyD88 pathway. Altogether the presented data indicate that the development of AHR and the induction of local inflammation might be the result of two parallel events, rather than one leading to another.

## Introduction

Respiratory infections, both viral and bacterial, are known to promote exacerbation and airway hyperresponsiveness (AHR) in asthmatic patients [1], [2], [3], [4]. The AHR development is often considered as a result of the inflammatory response, but the mechanisms behind this are poorly understood. It has become increasingly clear that activation of the innate immune system constitutes a critical element in the process [5]. Toll-like receptors (TLRs) are, as a part of the innate immune system, key elements in recognizing viral and bacterial components. Detection of microbes by TLRs evokes an inflammatory response. We, along with several other research groups, have demonstrated functionally active TLRs in nearly all cell types implicated in the pathogenesis of asthma and allergic airway disease, including eosinophils, neutrophils, mast cells, monocytes, dendritic cells, macrophages, B-cells, T-cells, epithelial cells and smooth muscle cells [6], [7], [8], [9], [10], [11]. Accordingly, several TLRs and TLR ligands have been associated with the development of asthma [12].

Epithelial and smooth muscle cells are the first to encounter invading microbes. Cultured human airway smooth muscle cells express several TLRs, with a pronounced expression of TLR3 and TLR4 [13]. Polyinosinic polycytidylic acid (poly(I:C)), a synthetic analogue of viral double-stranded RNA, is a ligand to TLR3 and lipopolysaccharide (LPS), a major component of the external membrane of gram-negative bacteria, is a ligand to TLR4. Poly(I:C) and LPS enhance airway smooth muscle contractions *in vitro* [13], [14], [15]. It is also well known that inflammatory mediators such as tumor necrosis factor alpha (TNF-α), interleukin-1 beta (IL-1β) and interferon gamma (IFN-γ) have the ability to up-regulate the TLR expression in airway smooth muscle cells. Further, if these cell are stimulated with poly I:C or LPS they increase their expression of several key immunomodulatory proteins like IL-6, eotaxin (CCL11), IL-8 (CXCL8), interferon gamma-induced protein 10 kDa (IP-10: CXCL10) and inter-cellular adhesion molecule 1 (ICAM-1) [16].

The present study was designed to explore the relation between infection and the development of AHR. To that end, mice were treated intranasal with either poly(I:C) or LPS followed by assessment of airway resistance in response to methacholine (MCh). Cells and cytokine levels in broncheoalveolar lavage fluid (BALF) were evaluated and MyD88-deficient C57BL/6 mice were used for further characterisation of pathways involved.

## Materials and Methods

### Animals

Female BALB/c and MyD88 deficient C57BL/6 mice (approximately 20 g, 8–12 weeks old) were obtained from Charles River (Sulzfeld, Germany) and Department of Microbiology, Tumor and Cell biology (MTC) at the Karolinska Institutet (Stockholm, Sweden) respectively. The mice were housed in a conventional animal house with 12-hours dark/light cycles and kept in plastic cages with adsorbent bedding material. Water and pelleted food were provided *ad libitum*. All animal procedures were approved by the local ethics committee at Karolinska Institutet (Stockholm norra djurförsöksetiska nämnd). Ethical permit numbers 152/06 and 348/11.

### Treatment protocol

On days 1 to 4 the animals were anaesthetized by isoflurane inhalation and 20 µl of PBS, 0.1 mg/ml LPS from Escherichia coli 0127:B8 (Sigma-Aldrich, St Louis, MO, USA), or 1 mg/ml lyophilized poly(I:C) (Invivogen, San Diego, CA, USA) were administered intranasally. Lung mechanics together with collection of BALF were assessed at day 5, i.e. 24 h after the last intranasal treatment.

### Lung mechanics

Mice were ventilated with the flexiVent™ animal ventilator (Scireq, Montreal, Canada). Preparations included anaesthesia (i.p. pentobarbital sodium, 90 mg·kg^−1^·bw) and tracheostomy (18-gauge cannula). Animals were put on a heating pad (body temperature, 37°C) and connected to the ventilator. After ventilation was started, bilateral thoracotomy was performed, in order to equalize pleural pressure to atmospheric and to exclude chest wall contribution to the mechanics. The ventilation frequency was 2.5 Hz in a quasi-sinusoidal fashion and by this mode the pressure waveform was sinusoidal during inflation. The tidal volume was 12 ml·kg^−1^ body weight and the positive end-expiratory pressure (PEEP) was 3 cm H_2_O. An i.v. catheter was inserted into the tail vein. Four sigh manoeuvres to three times the tidal volume were performed in order to stabilize the baseline lung resistance. After a five minutes resting period, Acetyl-ß-methylcholine (Sigma-Aldrich, St Louis, MO, USA) was injected through the tail vein in increasing doses (0.01, 0.03, 0.1, 0.3, 1 and 3 mg·kg^−1^·body weight), in order to induce AHR. Lung resistance (R_L_) and lung compliance (C_L_) were measured by assuming a single-compartment linear model and multiple linear regressions. Changes in reactivity and sensitivity were assessed using non-linear regression analysis to calculate the maximum responses (E_max_) and effective dose for half maximal response (ED_50_).

### Broncheoalveolar lavage

BAL was performed directly after lung function measurements. 1 ml PBS containing 0.6 mM ethylendiaminetetraacetic acid (EDTA) was lavaged three times in the lung. The fluid was centrifuged at +4°C, 1200 rpm, for 10 minutes and the supernatant was stored at −80°C until use. Lysis buffer (150 mM NH4Cl, 10 mM KHCO3, 0.1 mM EDTA, pH 7.2), was used for 2 min, to lyse the red blood cells, and followed by washing in PBS. Total cell number was counted and expressed as cells·ml^−1^ BALF. Differential cell counts were performed on May-Grünwald/Giemsa stained cytospins, counting a minimum of 300 cells, in a blinded manner.

### Measurement of inflammatory mediators

Cytokines in BALF were measured using the Cytokine Mouse 20-Plex Panel together with RANTES Mouse Singleplex Bead Kit (Invitrogen Corp., Carlsbad, CA, USA) that were run in a Luminex200 system. The simultaneous immunoassay was carried out according to instructions of the manufacturer. In brief, specific antibodies labelled with spectrally encoded beads were applied to the samples and standards. After incubation, the beads were washed and mixed with specific biotinylated detector antibodies. Subsequently, excess of biotinylated antibodies was removed by a washing step followed by addition of Streptavidin-R-phycoerythrin (RPE), in order to conjugate and label the detector antibodies. By monitoring the spectral properties of the beads and the amount of associated RPE flourescence by the Bio-Plex System (Bio-Rad Laboratories, Hercules, CA, USA) the concentration of one or more cytokines could be determined. The cytokines measured were: fibroblast growth factor (FGF basic, limit of detection (lod) 101 pg/mL), granulocyte macrophage colony-stimulating factor (GM/CSF, lod 10 pg/mL), IFN-γ (lod 1 pg/mL), IL-1α (lod 10 pg/mL), IL-1β (lod 8,7 pg/mL), IL-2 (lod 10 pg/mL), IL-4 (lod 5 pg/mL), IL-5 (lod 6,5 pg/mL), IL-6 (lod 2,7 pg/mL), IL-10 (lod 120 pg/mL), IL-12 (lod 11 pg/mL), IL-13 (lod 20 pg/mL), IL-17 (lod 5 pg/mL), IP-10 (lod 46 pg/mL), keratinocyte chemoattractant (KC: CXCL1, lod 11,5 pg/mL), monocyte chemotactic protein-1 (MCP-1: CCL2, lod4,2 pg/mL), monokine induced by gamma interferon (MIG: CXCL9, lod 3 pg/mL), macrophage inflammatory protein-1 α (MIP-1α: CCL3, lod 15,2 pg/mL), TNF-α (lod 4,5 pg/mL), vascular entothelial growth factor (VEGF, lod 10 pg/mL9) and RANTES (Regulated upon Activation Normal T-cell Expressed and Secreted: CCL5, lod 60 pg/mL).

### Lung histology

Lungs were removed and immersed with 4% buffered formaldehyde. They were then embedded in paraffin, sectioned and stained in haematoxylin and eosin. Histological findings such as peribronchial, parenchymal and perivascular cell infiltration were semi-quantitatively graded in a blinded manned as 0 to 5 (0 = no cell infiltration, 3 = moderate, 5 = abundant), with a focus on inflammatory cells.

### Statistical analysis

Data were analyzed using Graph Pad Prism, version 5.01, software (GraphPad Software Inc. San Diego, CA, USA). Results are presented as mean ±standard error of mean (SEM) and n equal's number of subjects. For comparison of data, one-way analysis of variance (ANOVA) was employed and if significant the data was further analyzed by Bonferroni post hoc test. ED_50_ was analysed as log-values to follow normal distribution. A p-value of 0.05 or less was considered statistically significant.

## Results

### Airway hyperresponsiveness

**Figure 1:**
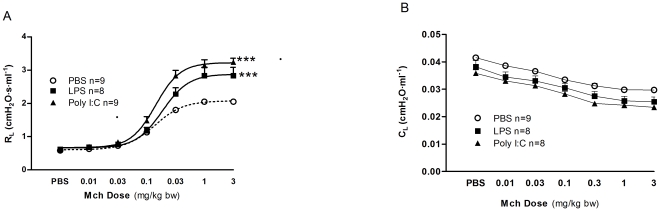
LPS and poly(I:C) induced airway hyperresponsiveness. (A) Changes in lung resistance (R_L_) and (B) compliance (C_L_) in response to methacholine in BALB/c mice treated during 4 days with vehicle (PBS), poly(I:C) or LPS i.n.. Results are expressed as mean ± SEM. *p<0.05, ***p<0.001 vs. PBS.

Airway function was assessed in three treatment groups, vehicle (PBS), poly(I:C) and LPS, before and during intravenous injections of increasing concentrations of MCh. In BALB/c mice R_L_ values did not differ between the groups during the initial baseline period. In the poly I:C group, a decrease in C_L_ compared to vehicle was found at baseline (p<0.05), in the other groups no baseline difference in C_L_ was registered. All three groups responded to MCh with an increase in R_L_ and a decrease in C_L_. The maximal resistance (R_Lmax_) in the poly(I:C) and the LPS treated groups, were higher than in the control group (PBS: R_Lmax_ 2.07±0.11 cm H_2_O·s·ml^−1^, poly I:C: R_Lmax_ 3.32±0.12 cm H_2_O·s·ml^−1^; p<0.001 and LPS: R_Lmax_ 2.87±0.25 cm H_2_O·s·ml^−1^; p<0.01) (Figure 1A). For the highest MCh dose, the C_L_ was decreased in both the poly I:C and LPS groups (p<0.001, p≤0.05 respectively) (Figure 1B). No differences in ED_50_ levels were seen (PBS: ED_50_ 0.17±0.04 mg·kg^−1^ body weight, poly(I:C): ED_50_ 0.18±0.04mg·kg^−1^ body weight, LPS: ED_50_ 0.19±0.03 mg·kg^−1^ body weight).

The C57BL/6 mice did not develop AHR, regardless of the treatment given.

### Inflammatory response

**Figure 2:**
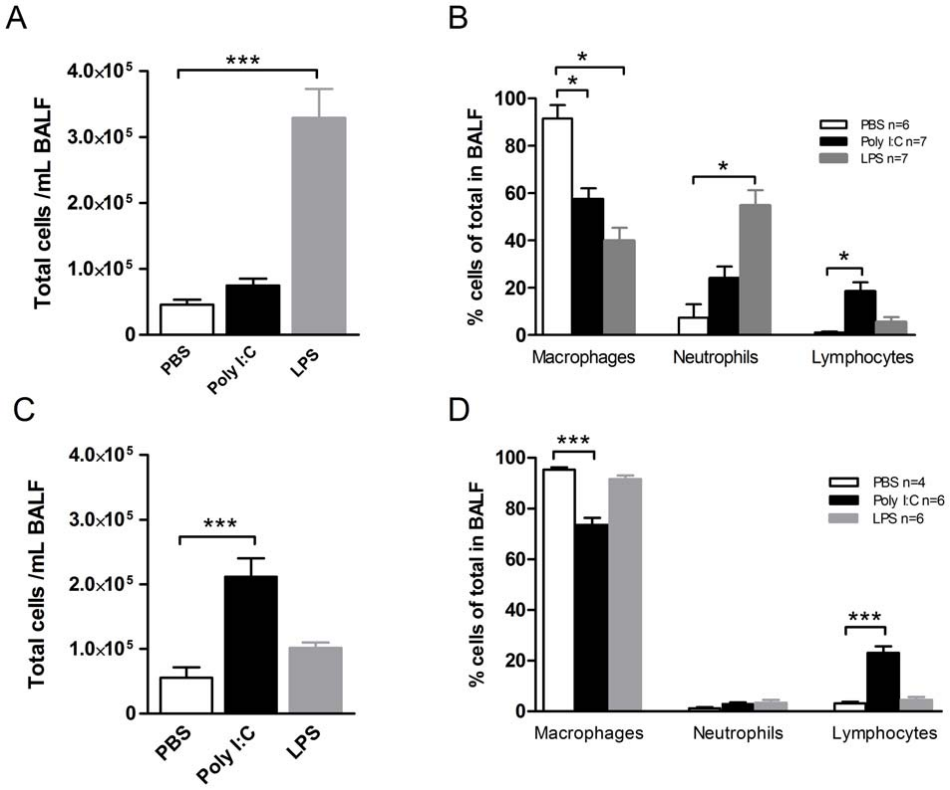
Poly(I:C) and LPS induced cell recruitment in BALF. (A, C) Absolute and (B, D) relative cell numbers in BALF in BALB/c mice (A, B) respectively MyD88deficient C57BL/6 mice (C, D) treated during 4 days with vehicle (PBS), poly(I:C) or LPS i.n.. Results are expressed as mean ± SEM. *p<0.05.

BALF was collected in order to analyze cells and cytokines. In BALB/c mice LPS treatment exhibited an increase in total cell count in comparison with control animals (LPS: 33.0±4.4×10^4^ cells, PBS: 4.6±0.79×10^4^ cells; p<0.001), whereas this was not seen for poly(I:C) (poly(I:C): 7.5±1.0×10^4^ cells; Figure 2 A). Examining individual cell types, LPS induced an increased proportion of neutrophils whereas an increase of lymphocytes was seen after poly(I:C) treatment (Figure 2 B). A different cell distribution was found in MyD88 deficient mice, with a significant increase in the total cell count after poly(I:C) treatment (poly(I:C): 21.0±7.1×10^4^ cells, PBS: 5.5±3.7×10^4^ cells; p<0.001), whereas this was not seen following LPS (10.2±1.8×10^4^ cells; Figure 2 C). An increased proportion of lymphocytes was found after poly(I:C) treatment (Figure 2 D).

**Figure 3:**
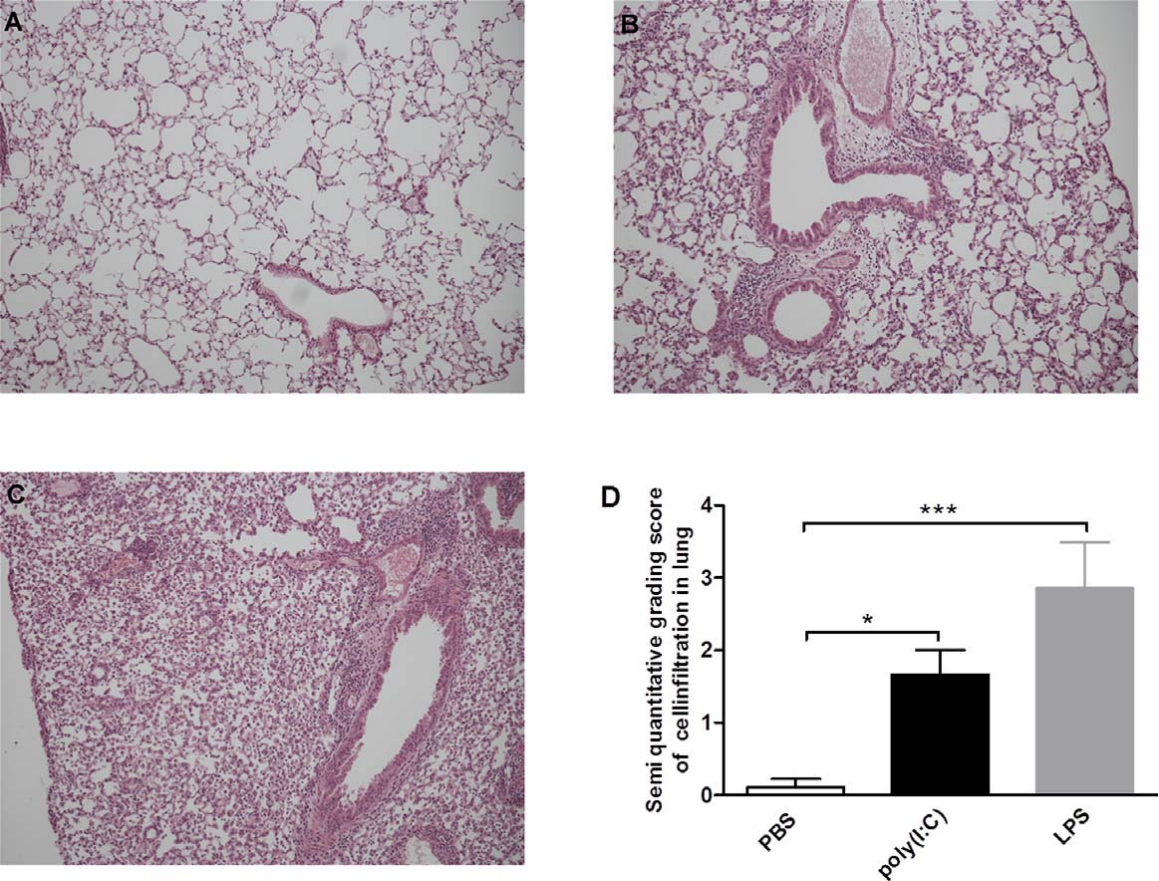
Poly(I:C) and LPS induced cell recruitment in lung tissue. Haematoxylin and eosin stained cells in BALB/c mice treated i.n. during 4 days with (A) vehicle, (B) poly(I:C) (mainly neutrophils) and (C) LPS (mainly lymphocytes). Magnification is ×200. (D) Semi quantitative grading score of cell infiltration in the lung.

In the BALB/c mice LPS also induced an increased infiltration of inflammatory cells, mainly neutrophils, in the lung tissue, with a distribution of cells in the peribronchial, perivascular area and also within the parenchyma. Infiltration was demonstrated as a semi quantitative score from 0 to 5, where 0 = no infiltration (PBS: 0.1±0.1, LPS: 2.9±0.6; p≤0.001). Poly(I:C) treatment induced an inflammatory cell influx with predominantly lymphocytes, mainly in the peribronchial and perivascular areas (poly(I:C): 1.7±0.3; p≤0.05) (Figure 3). Differentiation of neutrophils and lymphocytes was made on their morphologic appearance at a high magnification (×1000).

**Figure 4:**
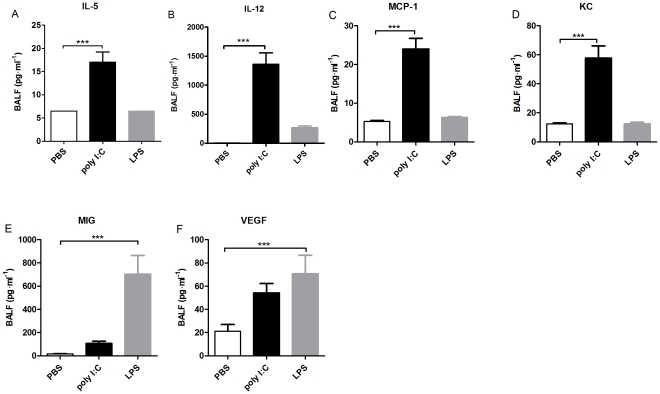
Poly(I:C) and LPS induced cytokine release in BALF. Levels of (A) IL-5, (B) IL-12, (C) MCP-1, (D) KC, (E) MIG and (F) VEGF in BALB/c mice treated during 4 days with vehicle (white), poly(I:C) (black) or LPS (gray) i.n. Results are expressed as mean ± SEM. PBS n = 10; poly I:C n = 12; LPS n = 13; ***p<0.001.

In order to further dissect potential differences between poly(I:C) and LPS induced inflammation, 21 cytokines and growth factors were measured in BALF. The levels of IL-5 IL-12, MCP-1 and KC were increased in the poly I:C treated group (IL-5: PBS: 6.5±0.0, poly I:C: 17.0±2.2, p≤0.001, IL-12: PBS: 11±0.0, poly I:C: 1360.0±197.6, p p≤0.001, MCP-1: PBS: 5.3±0.2, poly I:C: 24.0±2.8, p≤0.001, KC: PBS: 12.4±0.6, poly I:C: 57.7±8.5, p≤0.001), whereas MIG and VEGF were increased after LPS treatment (MIG: PBS: 16.9±0.7, LPS: 702.1±60.5, p≤0.001, VEGF: PBS: 21.1±5.8, LPS: 70.7±15.9, p≤0.001) (Figure 4). No significant differences between the treatment groups where found for IL-1α, IL-17, MIP-1α, 1L-2, IL-6, IL-13, IP-10, RANTES or TNF-α. No detectable levels were found for FGF basic, GM/CSF, IFN-γ, IL-1β, IL-4, IL-10 or MIP-1α.

## Discussion

The treatment of BALB/c mice with poly(I:C) and LPS during 4 consecutive days resulted in the development of AHR in conjunction with a local inflammatory reaction. The degree of AHR was essentially the same irrespectively of which TLR receptor that was stimulated, whereas the inflammatory response appeared to be receptor specific. Poly(I:C) caused a limited cell influx in BALF characterized by an increased proportion of lymphocytes and secretion of IL-5, IL-12 MCP-1 and KC whereas LPS induced a marked inflammation dominated by neutrophils together with MIG and VEGF. The neutrophil influx appeared to be mediated through a MyD88 dependent pathway.

The presented report is the first to compare the effects of repeated exposure during four consecutive days to LPS and poly(I:C) on airway resistance. However, acute experiments demonstrating that LPS [13], [14], poly(I:C) [17] and influenza A (activates TLR3) [18] all can induce AHR have been performed. We have in a previous study of isolated murine airways demonstrated that 4 days of treatment with LPS and poly(I:C) resulted in a marked increase in the contractile response to both bradykinin and [des-Arg^9^]-bradykinin [19]. These findings support the present AHR data. It is known that both bradykinin B1 and B2 receptors can contribute to the allergen induced AHR in rats [20]. Since both TLR3 and TLR 4 are expressed on airway smooth muscle cells, the presently demonstrated *in vivo* effects of poly(I:C) and LPS can be explained by a direct activation of the airway smooth muscle. [19]. However, the possibility of an indirect effect has also to be considered. Such a response could be mediated via the release of inflammatory mediators from the bronchial epithelium and/or inflammatory airway cells like macrophages, neutrophils and lymphocytes. All of these cells are known to express a variety of TLRs including TLR3 and TLR4 [6], [8], [10].

The present investigation revealed that LPS given to BALC/c mice caused a severe inflammatory reaction with a marked influx of neutrophils, whereas the poly(I:C) treatment resulted in a limited lymphocyte invasion. This is well in line with the literature demonstrating TLR4 to have ample capacity to facilitate leukocyte migration [21], [22], and poly(I:C) (in the concentration used) to lack the same effect [17]. It is therefore tempting to conclude that AHR can be developed without correlation to the recruitment of a specific set of leukocytes. There are in fact an increasing number of studies supporting that AHR and airway inflammation may be dissociated [21], [22], patting focuses on local events in the ASM. Since the same inflammatory mediators could be released from a variety of cells this assumption does not exclude the possibility of cytokine/chemokine mediated indirect effect on the airway smooth muscle [23], [24]. We therefore investigated a set of 21 inflammatory mediators in BALF in BALB/c mice treated with LPS, poly(I:C) and vehicle. Poly(I:C) increased the presence of IL-5, IL-12, MCP-1 and KC, all known to related to viral stimulation and lymphocyte activation [25], [26]. LPS induced secretion of MIG and VEGF, involved in neutrophil recruitment and remodelling, associating LPS to bacterial infections. However, some of our findings were not so straight forward signifying the complex nature of *in vivo* models, where we like in the present experiment, only have a single time point (at the end of the experiment) for evaluation a dynamic events known to change over time. Secondary and tertiary events might explain why, at the end of the experiment, increased levels of the monocyte chemoattractant MCP-1 [27] can be found together with a reduced number of macrophages. This may also explain how KC, a neutrophil chemoattractant, could be increased after poly(I:C) without remaining signs of neutrophil recruitment in BALF and vice versa for LPS [28]. No mediators were found to be increased by both TLR3 and TLR4 stimulation. On the contrary, the cytokine release appeared to be closely related to cells recruited.

TLR4 activates two distinct signalling pathways, one dependent on adaptors MyD88 and TIRAP (Toll/interleukin-1-receptor (TIR)-domain-containing adaptor protein). The other pathway, through TRIF (TIR-domain-containing adapter-inducing interferon-β), is MyD88 independent. The TRIF pathway is also shared by TLR3. Hence the use of MyD88 deficient mice can make it possible to distinguish activation caused by TLR4 from those induced by TLR3. Even though BALB/c mice are known to be preferred for studies of inflammatory induced AHR, MyD88 deficient mice on BALB/c background are not available. C57BL/6 mice are recognized to exhibit a low degree of inflammation-induced AHR but has similar antigen induced cellular levels in BALF as the BALB/c strain [29], [30]. It was therefore expected that, the repeating of the protocol using the MyD88 deficient C57BL/6 mice, should cause no AHR development, However, poly(I:C) caused a raised cell influx in BALF, something that was not seen after LPS. The low cell count after LPS treatment indicates that the inflammation is a result of MyD88 dependent TLR4 signalling and this differs from TLR3, where inflammation seems to be an effect of MyD88 independent signalling probably through the TRIF pathway. The absence of AHR in the C57BL/6 mice, to at least poly(I:C), is in accordance with the lower increase in smooth muscle reactivity to poly(I:C) in C57BL/6 than BALB/c mice [30], and further argument for that the TLR-induced AHR is mediated through an effect directly on the smooth muscle.

Acute exacerbations, often seen in association with infection, are a major cause of morbidity in asthma [25], [26]. Far from all asthma patients benefit from the existing therapeutic alternatives [31]. A better knowledge of the role of infection in exacerbations might open the way for new therapeutic interventions. In the present study poly(I:C) activating TLR3 and LPS triggering TLR4, were chosen to represent viral and bacterial induced interactions, respectively. As demonstrated, both types of infections caused AHR, but the local inflammatory profiles characterized by inflammatory cell recruitment and cytokine release appeared to be receptor specific. This challenge the idea that inflammation is prerequisite for AHR development and suggests that at least part of the effect might be due to a direct microbial interaction with the smooth airway musculature [13].
